# Down-Regulated NOD2 by Immunosuppressants in Peripheral Blood Cells in Patients with SLE Reduces the Muramyl Dipeptide-Induced IL-10 Production

**DOI:** 10.1371/journal.pone.0023855

**Published:** 2011-08-19

**Authors:** Shui-Lian Yu, Chun-Kwok Wong, Purple Tsz-Yan Wong, Da-Peng Chen, Cheuk-Chun Szeto, Edmund K. Li, Lai-Shan Tam

**Affiliations:** 1 Department of Medicine and Therapeutics, Prince of Wales Hospital, The Chinese University of Hong Kong, Hong Kong, China; 2 Department of Chemical Pathology, Prince of Wales Hospital, The Chinese University of Hong Kong, Hong Kong, China; 3 Department of Clinical Laboratory, Children's Hospital of Chongqing Medical University, Chongqing, China; University of Nebraska – Lincoln, United States of America

## Abstract

**Background:**

Pattern recognition receptors (PRRs) such as Toll-like receptors are aberrantly expressed of peripheral blood mononuclear cells (PBMCs) in systemic lupus erythematosus (SLE) patients, for playing immunopathological roles.

**Methodology/Principal Findings:**

We investigated the expression and function of the PRR nucleotide-binding oligomerization domain (NOD2) in SLE. NOD2 expression in T, B lymphocytes, monocytes, myeloid dendritic cells (mDCs) and plasmacytoid dendritic cells (pDCs) was assessed in SLE patients and healthy controls (HCs) using flow cytometric analysis. *Ex vivo* production of cytokines from PBMCs upon NOD2 agonist muramyl dipeptide (MDP) stimulation was assessed using Cytometric Bead Array. Over-expression of NOD2 in monocytes was observed in immunosuppressant naïve SLE patients, and was positively associated with longer disease duration. Immunosuppressive therapy was an independent explanatory variable for downregulating NOD2 expression in CD8+ T, monocytes, mDCs and pDCs. *Ex vivo* basal productions of cytokines (IL-6, IL-8 and IL-10) were significantly increased in immunosuppressant naïve patients and patients with active disease despite immunosuppressants compared with HCs. Upon MDP stimulaiton, relative induction (%) of cytokines (IL-1β) from PBMC was significantly increased in immunosuppressant naïve patients with inactive disease, and patients with active disease despite immunosuppressant treatment compared with HCs. Immunosuppressant usage was associated with a decreased basal production and MDP induced relative induction (%) of IL-10 in patients with inactive disease compared with immunosuppressant naïve patients and HCs.

**Conclusions/Significance:**

Bacterial exposure may increase the NOD2 expression in monocytes in immunosuppressant naïve SLE patients which can subsequently lead to aberrant activation of PBMCs to produce proinflammatory cytokines, implicating the innate immune response for extracellular pathogens in the immunopathological mechanisms in SLE. Immunosuppressant therapy may downregulate NOD2 expression in CD8+ T lymphocytes, monocytes, and DCs in SLE patients which subsequently IL-10 reduction, contributing towards the regulation of immunopathological mechanisms of SLE, at the expense of increasing risk of bacterial infection.

## Introduction

Systemic lupus erythematosus (SLE) is an autoimmune disease with unknown etiology affecting more than one million individuals each year. It is characterized by B and T cell hyperactivity, and defects in the clearance of apoptotic cells and immune complexes [1]. Besides genetic factors, environmental triggers can also contribute to pathogenesis of SLE. An infectious etiology of SLE has been longstanding hypothesis [2], [3], [4] and with the discovery of pattern recognition receptors (PRRs) in SLE patients, the role of bacteria and viruses in the pathogenesis of SLE has been invigorated.

PRRs can alert and activate the innate immune system through recognizing the conserved molecular patterns to distinguish extrinsic pathogen-associated molecular patterns (PAMPs) and endogenous danger-associated molecular patterns (DAMPs). PRRs were involved in the development of various inflammatory disorders [5]. The activation of PRR-mediated proinflammatory cascades leads to the clearance of invading pathogen, and the subsequent resolution of inflammation [6]. Along with the identification of endogenous ligands for many PRRs, PRRs might also respond to damaged tissue and apoptotic cell debris [6]. Furthermore, activation of PRRs has been found to modulate adaptive immune responses through the regulation of co-stimulatory molecules, maturation of dendritic cells (DCs) and stimulation of B cells [7], [8]. Being the most studied PRRs, toll-like receptors (TLRs) mediated intracellular signaling is a crucial link between innate and adaptive immunity [9]. Peripheral blood mononuclear cells (PBMCs) of SLE patients with a higher expression of TLRs are more prone to be activated by diverse TLR ligands when compared with HCs [7], [10]. Signaling through MyD88- and Toll-IL-1 receptor-domain-containing adapter-inducing interferon-β (TRIF)-dependent pathways results in the activation of type I interferons (IFN)s, resulting in inflammation and subsequent tissue damage in SLE [7], [10], [11].

In contrast to the well elucidated membrane-bound TLRs, cytoplasmic nucleotide binding oligomerisation domain (NOD) receptors are a new family of PRRs for the recognition of extracellular PAMPs [12], [13]. Two NOD-like receptor (NLR) proteins, namely NOD1 and NOD2, can participate in the signaling events triggered by host recognition of specific motifs of bacterial peptidoglycans (PGNs) and, upon activation, induce the production of proinflammatory mediators [12]. NOD1 recognizes products from gram-negative bacteria (diaminopimelic acids), whereas NOD2 senses muramyl dipeptide (MDP), a peptidoglycan derived peptide from gram-negative as well as gram-positive bacteria [14]. It has been shown that NLRs complement and synergize with TLRs in innate immune responses [5], [6], [15], [16]. NLRs are associated with inflammatory bowel disease such as Crohn's disease (CD) [17]. However, the precise mechanisms by which NOD-mediated recognition of PGNs in the pathogenesis of inflammatory diseases are still unclear. Possible pathogenic mechanisms in rheumatoid arthritis (RA) patients and murine experimental models included [6], [16] (1) NOD2 functions to induce proinflammatory effects in the inflamed joint, whereas NOD1 has mainly an inhibitory role, exemplified by the inhibition of cytokine production and decrease in cell influx in a model of streptococcal cell wall (SCW)-induced arthritis; (2) NOD2 act synergistically with TLR in the production of proinflammatory and destructive mediators in RA patients. A better understanding of the intracellular events induced by the interaction between NLR and PGNs is therefore crucial for the elucidation of the mechanisms of both the recognition of pathogens by the innate immune system and the pathogenesis of chronic inflammation.

Apart from the putative link between genetic mutations of NOD2 and SLE [18], [19], [20], [21], [22], [23], little is known regarding the expression and function of NOD2 in SLE. Therefore, in this cross-sectional study, we investigated the role of NOD2 in the peripheral antigen presenting cells (APCs), T, B lymphocytes, as well as the role of NOD2 in the modulation of proinflammatory cytokines induced by NOD2 ligand MDP of SLE patients and control subjects.

## Methods

### SLE patients, control subjects, and blood samples

Forty-seven SLE patients of Southern Chinese origin were recruited at the Rheumatology Out-Patient Clinic of the Prince of Wales Hospital, the Chinese University of Hong Kong. Diagnosis of SLE was established according to the 1997 American College of Rheumatology (ACR) revised criteria for the classification of SLE.[24] Patients were excluded from the study if they had prior treatment with a monoclonal antibody or other biologic agents. SLE patients were divided into 3 groups according to disease activity as reflected by the systemic lupus erythematosus disease activity index (SLEDAI) [25] and the use of immunosuppressants as list below: Group 1) Patients with inactive disease (SLEDAI <4) who were never treated with immunosuppressants since the diagnosis or within the past 10 years, whichever longer; Group 2) Patients with inactive disease (SLEDAI <4) who had received or are currently on immunosuppressants; Group 3) Patients with active disease (SLEDAI >4) who had received or are currently on immunosuppressants. Thirty-one age- and sex-matched healthy Chinese volunteers were recruited as controls (HC group). Twelve mls of ethylenediamine tetra-acetic acid (EDTA) venous peripheral blood were collected from each patient and control.

Ethics approval has been obtained from Ethics Committee of The Chinese University of Hong Kong-New Territories Ease Cluster Hospitals, and informed consent was obtained from all participants according to the Declaration of Helsinki.

### Clinical and laboratory parameters

Patient information with regard to demographic characteristics, clinical features, serological profile and medications were retrieved from medical records. Physical examinations and laboratory investigations including complete blood count, liver and renal functions, levels of anti-double stranded DNA (dsDNA) titer, serum complements C3 and C4 levels were performed at study visit. SLEDAI [25] and Systemic Lupus International Collaborating Clinics score (SLICC) [26] were evaluated during clinical assessment. Serum level of anti-dsDNA was measured by in house ELISA using a calibration curve generated from six standards which were prepared with reference to the WHO international standard serum Wo80. The interassay coefficient of variation of the immunoassay for low and high controls was 14.7 and 6.7%, respectively. Serum C3 and C4 levels were determined by immunonephelometry (Immage 800; Beckman Coulter, California, USA). *Flare* is defined as increase in SLEDAI score by 3 or more compared to the last clinical visit [27], [28]. Major organ involvement was defined as the involvement of one or more of the following organs: the central nervous system, kidney, lung, heart and the hematologic system (hemolytic anemia, platelet <100,000/µL). Immunosuppressive agents included prednisolone, hydroxychloroquine (HCQ), azathioprine (AZA), cyclophosphamide (CYC) (oral or IV), cyclosporin A and mycophenolate mofetil (MMF) were prescribed for SLE patients.

### Flow cytometric analysis for T, B lymphocytes, monocytes and dendritic cells

PBMCs from SLE patients and HCs were purified by Ficoll Plus gradient centrifugation (GE Healthcare Life Sciences, NJ, USA). R-phycoerythrin (PE)-conjugated CD4, peridinin chlorophyll protein (PerCP)-conjugated CD8 and allophycocyanin (APC)-conjugated CD19 antibodies were purchased from BD Pharmingen Corp. for the identification of the CD4^+^ T, CD8^+^ T and CD19^+^ B lymphocyte sub-populations. Peripheral blood monocytes were analyzed by excluding lymphocytes using their forward- and side-scatter properties [7].

For the analysis of DCs, fluorescein iso-thiocyanate (FITC)-conjugated mouse anti-human major histocompatibility complex (MHC) Class II DR monoclonal antibody was used for prior positive gating (Thermo Fisher Scientific, NH, USA). PE-conjugated anti-CD14 and CD16, APC-conjugated anti-immunoglobulin-like transcript (ILT)-3/CD85k/LIR5 (Beckman Coulter Inc, CA, USA) antibody and PerCP-conjugated anti-CD33 antibody purchased from BD Biosciences Pharmingen were used according to our previously established method [29]. The DC population is identified as CD14 and CD16 double negative and CD85k positive population. Myeloid DCs (mDCs) and plasmacytoid DCs (pDCs) were further identified by high and low CD33 expression, respectively.

### Flow cytometric analysis of the expression of NOD2 in T, B lymphocytes, monocytes and DCs

The expression of NOD2 of different immune cell types of SLE patients and control subjects was investigated by flow cytometry (BD FACSCalibur, BD Biosciences Corp, CA, USA) as previously described [7]. Serum-blocked PBMCs were fixed and permeabilised using Fix/Perm solution (BD Biosciences, Mississauga, ON, Canada) for the intracellular staining of NOD2. Unconjugated mouse anti-NOD2 antibody (Bio-legend Corp, CA, USA), corresponding mouse IgG1, κ isotypic control antibody (BD Pharmingen Corp., San Diego, CA, USA), together with a fluorescein iso-thiocyanate (FITC)-conjugated goat anti-mouse IgG (H+L) secondary antibodies (Zymed Laboratories, Inc., CA, USA), were used for intracellular staining. The expression of NOD2 in final cell suspension with 10,000 events was assessed using flow cytometry and the results were expressed as mean fluorescence intensity (MFI).

### *Ex vivo* induction of cytokines by NOD2 ligand

Aliquots of 1×10^5^ cells resuspended PBMCs in culture medium RPMI1640 supplemented with 10% fetal calf serum (Gibco Laboratories) were dispensed in each well of a 96-well plate (Nalge Nunc International, IL, USA). The culture medium used was free of detectable endotoxin (<0.1 EU/ml) and all other solutions were prepared using pyrogen-free water and sterile polypropylene plastic ware. The cells were then incubated with or without NOD2 ligand muramyl dipeptide (MDP) (Invivogen Corp, San Diego, CA, USA) at 0.5 µg/ml for 24 h at 37°C in a 5% CO2 atmosphere. The cell-free supernatant was harvested and stored at −70°C for subsequent assays of cytokines and chemokines.

### Assay of the induction of human cytokines by NOD2 stimulation

The basal concentration and induction of cytokines including interleukin (IL)-1β, tumour necrosis factor (TNF)-α, IL-6, IL-8 and IL-10 were measured simultaneously using human inflammatory cytokine Cytometric Bead Array (BD Pharmingen Corp., San Diego, CA, USA) using flow cytometry (FACSCalibur, BD Biosciences). Supernatant samples were analyzed using a multi-fluorescence BD FACSCalibur™ flow cytometer with BD CellQuest™ software and BD™ CBA software. The relative percentage inductions of cytokines compared with medium control was calculated based on the percentage difference of induction in medium and after NOD2 ligand stimulation. Relative induction of cytokine (%) was calculated by the equation, (induction of cytokine by NOD2 ligand activation - induction of cytokine of medium control)/induction of cytokine of medium control ×100%.

### Statistical analysis

Results were expressed as mean ± standard (SD) for normally distributed data. Non-normally distributed data were expressed as median (interquartile range, IQR). Chi-squared tests were used for categorical variables. For continuous variables, Student's *t*-test and Mann-Whitney U tests were used, where appropriate. Association between the potential explanatory variables and NOD2 expression were tested using Chi-square tests for categorical variables and Person or Spearman correlation for continuous variables with normal and skewed distribution, respectively. Variables with *p* value <0.05 in the univariate analysis were entered into linear regression analysis (stepwise, forward). Variables that were skewed were logarithmically transformed before entering the regression analysis. All hypotheses were 2-tailed, and *p* values <0.05 were consider significant. Analyses were performed using SPSS for Windows, version 13.0 (SPSS Inc., Chicago, IL, USA).

## Results

### Characteristics of SLE patients and control subjects

Forty-seven female Chinese SLE patients were recruited and divided into 1 of 3 SLE groups according to the current disease activity and the use of immunosuppressants as listed before (Group 1, 2 and 3: n = 17, 13 and 17 respectively). Thirty-one age-matched (36±12 years) female healthy Chinese volunteers were recruited as controls. Demographics and clinical characteristics were summarized in Table 1. The mean disease duration among the three SLE groups was similar. Group 1 had low cumulative inflammatory burden as indicated by a significantly lower damage index (SLICC) and a significantly lower prevalence of major organ involvement compared to Groups 2 and 3, and a low disease activity not requiring immunosuppressants. Current disease activity as reflected by the SLEDAI scores were significantly higher in Group 3 compared to Groups 1 and 2 according to the study design, and all patients in Group 3 had a recent flare of disease activity. Serologically, Group 3 patients had a significantly lower level of C3 and C4, and a higher prevalence of elevated anti-dsDNA antibody levels compared with Group 1. Clinical manifestations at the time of study included neuropsychiatric (4/47, 9%), nephritis (27/47, 57%), serositis (4/47, 9%), haematological (14/47, 30%) and arthritis (18/47, 38%). Cutaneous manifestations included discoid rash (3/47, 6%), malar rash (11/17, 23%), photosensitivity (7/47, 15%) and oral ulcers (3/47, 6%). Group 3 patients had a significantly higher prevalence of nephritis (17/17, 100%) and hematological involvement (9/17, 53%), as well as a significantly higher proportion of patients who had received MMF.

**Table 1:** Demographic and clinical characteristics of patients with systemic lupus erythematosus.

	SLE patients (n = 47)		
	Group 1(n = 17)	Group 2(n = 13)	Group 3(n = 17)
**Demographic characteristics**			
Sex (female/male)	17/0	13/0	17/0
Age at study, mean ± s.d. (range), year	42±15 (12–63)	44±10 (26–65)	36±12 (19–59)
			
**Clinical features**			
SLE duration, mean ± s.d. (range), year	9±5 (0–18)	8±7 (0–27)	8±6 (0–21)
SLICC	0 (0–0) &, *	1 (0–2)	0 (0–4)
SLEDAI score	0 (0–1) ***	0 (0–2) ##	6 (4–11)
Flare┼┼┼, no. (%)	0/17 (0%) ***	0/17 (0%) ###	17/17 (100%)
**Serological features**			
Serum complement C3, g/l	0.8 (0.8–1.1) **	0.7 (0.6–1.0) #	0.6 (0.4–0.8)
Serum complement C4, g/l	0.2 (0.2–0.3) **	0.2 (0.2–0.3) #	0.1 (0.1–0.2)
Elevated anti-dsDNA (>100 IU/ml), no. (%)	0/17 (0%) &&, ***	7/13 (54%)	14/17 (82%)
Anti-dsDNA titer, IU/ml	n.a.	341±239 (150–840)	383±271 (115–1000)
**Number of major organ involvement, n. (%)**	5/17 (29%) &&&, **	11/13 (85%)	17/17 (100%)
0	12/17 (71%) &&&, **	2/13 (15%)	0/17 (0%)
1	5/17 (29%)	7/13 (54%) #	2/17 (12%)
≥2	0/17 (0%) &, ***	4/13 (31%) ##	15/17 (88%)
**Clinical manifestation, n. (%)**			
Neuropsychiatric	4/47 (9%)		
	0/17 (0%)	1/13 (8%)	3/17 (18%)
Nephritis	27/47 (57%)		
	2/17 (12%) &&, ***	8/13 (62%) ##	17/17 (100%)
Serositis	4/47 (9%)		
	0/17 (0%)	1/13 (%)	3/17 (18%)
Hematologic	18/47 (38%)		
	3/17 (18%) *	6/13 (46%)	9/17 (53%)
**Immunosuppressive therapy ever, n (%)**	n.a.	13/13 (100%)	17/17(100%)
Prednisolone	n.a.	12/13 (92%)	16/17 (94%)
Hydroxychloroquine	n.a.	10/13 (77%)	13/17 (76%)
Azathioprine	n.a.	10/13 (77%)	10/17 (59%)
Cyclophosphamide (oral or IV)	n.a.	7/13 (54%)	4/17 (24%)
Cyclosporin A	n.a.	1/13 (8%)	2/17 (12%)
Mycophenolate mofetil	n.a.	1/13 (8%) #	7/17 (41%)

Values are median (interquartile range, IQR) unless stated otherwise; s.d., standard deviation; n.a., not applicable. SLE, systemic lupus erythematosus; Group 1) Patients with inactive disease (SLEDAI <4) who were never treated with immunosuppressants since the diagnosis or within the past 10 years, whichever longer; Group 2) Patients with inactive disease (SLEDAI <4) who had received or are currently on immunosuppressants; Group 3) Patients with active disease (SLEDAI >4) who had received or are currently on immunosuppressants. SLEDAI, systemic lupus erythematosus disease activity index; SLICC, Systemic Lupus International Collaborating Clinics Score;

┼ “Never” refers to SLE patients never be given treatment of immunosuppressants [prednisolone, hydroxychloroquine, azathioprine, cyclophosphamide (oral or IV), cyclosporin A and mycophenolate mofetil] since the diagnosis of SLE or within recent 10 years;

┼┼ “Ever” refer to use of immunosuppressants since the diagnosis of SLE.

┼┼┼ “*Flare*” is defined as increase in the SLEDAI score by 3 or more;

& *p*<0.05,

&& *p*<0.01,

&&& *p*<0.001, comparing between Group 1 and Group 2;

*p<0.05,

**p<0.01,

***p<0.001, comparing between Group 1 and Group 3;

# *p*<0.05,

## *p*<0.01,

### p<0.001, comparing between Group 2 and Group 3.

### Protein expression of NOD2 in T and B lymphocytes, monocytes and DCs

**Figure 1:**
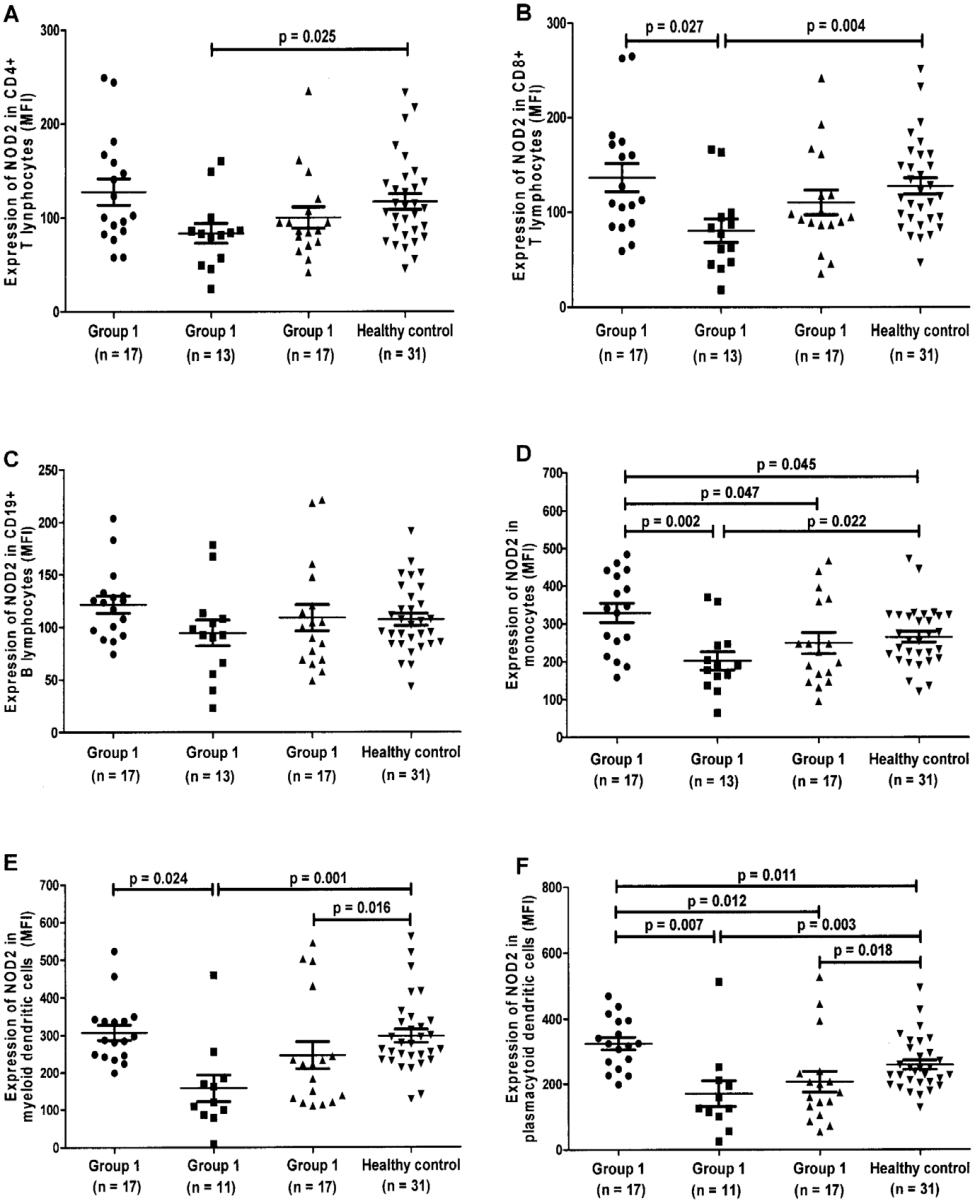
Expression of intracellular NOD2 in CD4^+^ T, CD8^+^ T, CD19^+^ B lymphocytes, monocytes, myeloid dendritic cells and plasmacytoid dendritic cells using flow cytometry. Group 1) Patients with inactive disease (SLEDAI <4) who were never treated with immunosuppressants since the diagnosis or within the past 10 years, whichever longer; Group 2) Patients with inactive disease (SLEDAI <4) who had received or are currently on immunosuppressants; Group 3) Patients with active disease (SLEDAI >4) who had received or are currently on immunosuppressants. The differential protein expression of intracellular pathogen recognition receptor NOD2 in (A) CD4+ T lymphocytes, (B) CD8+ T lymphocytes, (C) CD19+ B lymphocytes, (D) Monocytes, (E) myeloid dendritic cells (F) plasmacytoid dendritic cells of SLE patients and healthy controls by flow cytometry were shown as median (IQR) of mean fluorescence intensity (MFI) subtracting corresponding isotypic controls in scatter plots.

The differential protein expression of intracellular pathogen recognition receptor NOD2 in (A) CD4+ T, (B) CD8+ T, (C) CD19+ B, (D) Monocytes, (E) mDCs and (F) pDCs of SLE patients and HCs by flow cytometry were shown in scatter plots as average mean fluorescence intensity (MFI) (Figure 1).

### Effects of SLE on NOD2 expression compared to healthy controls

NOD2 expression in monocytes (Figure 1D, *p* = 0.045) and pDCs (Figure 1F, *p* = 0.011) were significantly increased in SLE patients with inactive disease who were not receiving immunosuppressive treatment (Group 1) when compared to HCs.

### Effects of immunosuppressants on NOD2 expression in SLE compared to healthy controls

For SLE patients who had been on immunosuppressants, NOD2 expression was significantly decreased in mDCs (Figure 1E, *p* = 0.016) and pDCs (Figure 1F, *p* = 0.018) of patients with active disease (Group 3) when compared to HCs. Similarly, significantly decreased NOD2 expression was observed in CD4+ T lymphocytes (Figure 1A, *p* = 0.025), CD8+ T lymphocytes (Figure 1B, *p* = 0.004), monocytes (Figure 1D, *p* = 0.022), mDCs (Figure 1E, *p* = 0.001), pDCs (Figure 1F, *p* = 0.003) of patients with inactive disease (Group 2) compared to HCs.

### Effects of immunosuppressants on NOD2 expression in SLE

For SLE patients with inactive disease (Groups 1 and 2), the expression of NOD2 were found to be significantly lower in CD8+ T (Figure 1B, *p* = 0.027), monocytes (Figure 1D, *p* = 0.002), mDCs (Figure 1E, *p* = 0.024), pDCs (Figure 1F, *p* = 0.007) in the group treated with immunosuppressants (Group 2) when compared to the non-treated group (Group 1). Moreover, NOD2 expression was decreased significantly in monocytes (Figure 1D, *p* = 0.047) and pDCs (Figure 1F, *p* = 0.012) in SLE patients with active disease despite on immunosuppressive treatment (Group 3) compared with those with inactive disease who were not receiving immunosuppressive treatment (Group 1). However, there was no significant difference in NOD2 expression between SLE patients on immunosuppressants with (Group 3) and without (Group 2) active disease (Figure 1A–F) (all *p*>0.05).

### Associations between clinical demographic variables, the use of immunosuppressants and the expression of NOD2

Tables 2, 3 and 4 summarized the association between clinical and treatment variables and the expression of NOD2 in the univariate analysis (Table 2: continuous variables, Table 3 and 4: categorical variables). There were positive correlations between disease duration and the expression of NOD2 in CD8+ T, CD19+ B and monocytes (all *p*<0.01) (Table 2). Significantly increased NOD2 expression in CD19+ B was found in patients with more than one major organ involvement compared with those without major organ involvement (118±51 MFI vs 93±31 MFI, *p*<0.05) (Table 3).

**Table 2:** Univariate analysis: Person or Spearman's correlation between the expression of NOD2 and clinical demographic variables in SLE patients (continuous variables).

	NOD2 expression											
	CD4+ T lymphocytes		CD8+ T lymphocytes		CD19+ B		Monocytes		mDC		pDC	
	n	r	n	r	n	r	n	r	n	r	n	r
**Clinical characteristics**												
SLE duration, year	47	0.287	**47**	**0.423** **	**47**	**0.486** **	**47**	**0.466** **	45	0.255	45	0.132
SLEDAI score	47	0.184	47	0.115	47	0.265	47	0.221	45	0.245	45	0.319
Serum C3, g/l	47	0.067	47	0.067	47	0.115	47	0.201	45	0.046	45	0.168
Serum C4, g/l	47	0.077	47	0.058	47	0.071	47	0.108	45	−0.019	45	0.095
Anti-dsDNA titer, IU/ml	21	0.221	21	−0.131	21	−0.400	21	−0.378	20	−0.031	20	−0.076
**Immunosuppressive therapy**												
Prednisolone												
Current dose, mg	29	−0.125	29	−0.047	29	−0.043	29	−0.019	27	0.206	27	−0.095
Cumulative dose, g	29	0.018	29	−0.096	29	0.156	29	−0.105	27	−0.017	27	−0.015
Hydroxychloroquine												
Current dose, mg	19	0.223	19	0.280	19	0.109	19	0.351	18	0.586	18	0.177
Cumulative dose, g	23	0.320	23	0.008	23	0.045	23	0.058	22	0.203	22	0.001
Azathioprine												
Current dose, mg	7	−0.532	7	−**0.808** *	7	−0.315	7	−0.611	6	−0.440	6	−0.371
Cumulative dose, g	20	−0.147	20	−0.087	20	−0.138	20	−0.045	**18**	−**0.560** *	18	0.041
Cyclophosphamide (oral or IV)												
Current dose, mg	6	−0.949	6	−0.633	6	−0.633	0	−0.633	6	−0.791	6	−0.649
Cumulative dose, g	11	0.529	11	0.242	11	0.295	11	−0.258	9	0.333	9	−0.171
Cyclosporin A												
Current dose, mg	1	n.a.	1	n.a.	1	n.a.	1	n.a.	1	n.a.	1	n.a.
Cumulative dose, g	3	n.a.	3	n.a.	3	n.a.		n.a.	3	n.a.	3	n.a.
Mycophenolate mofetil												
Current dose, mg	0	n.a.	0	n.a.	0	n.a.	0	n.a.	0	n.a.	0	n.a.
Cumulative dose, g	8	−0.667	8	0.238	8	0.143	8	−0.489	8	−0.119	8	−0.289

mDC, myeloid dendritic cells; pDC, plasmacytoid dendritic cells; *r*  =  Person or Spearman's correlation coefficient;

*p<0.05,

**p<0.01.

**Table 3:** Univariate analysis: the relationship between clinical characteristics, the use of drugs ever and the expression of NOD2 in SLE patients (categorical variables).

	NOD2 expression											
	CD4+ T lymphocytes				CD8+ T lymphocytes				CD19+ B lymphocytes			
	Yes		No		Yes		No		Yes		No	
	n		n		n		n		n		n	
**Clinical characteristics**												
Flare ┼	17	100±46	30	109±54	17	110±53	30	112±60	17	109±52	30	110±40
Elevated anti-dsDNA(>100 IU/ml)	21	93±44	26	116±55	21	100±53	26	121±60	21	102±49	26	116±40
Major organ involvement ≥1	32	103±41	15	111±69	32	109±48	15	111±69	**32**	**118**±**51** *	**15**	**93**±**31**
**Clinical manifestations**												
Neuropsychiatric	4	110±86	43	105±48	4	113±89	43	111±56	4	112±76	43	109±41
Nephritis	27	99±41	20	114±62	27	104±49	20	122±67	27	109±46	20	110±43
Serositis	4	136±67	43	103±49	4	134±73	43	110±56	4	147±57	43	106±42
Hematologic	18	114±47	29	101±54	18	119±50	29	107±62	18	126±48	29	99±39
**Immunosuppressive therapy** **ever** ┼┼	**30**	**93**±**43** *	**17**	**128**±**58**	**30**	**98**±**51** *	**17**	**136**±**61**	30	103±48	17	122 ±34
Prednisolone	29	95±41	18	122±61	29	100±50	18	130±60	29	106±47	18	116±40
** **Hydroxychloroquine	23	100±46	24	111±56	23	105±55	24	118±60	23	110±56	24	109±35
Azathioprine	20	103±43	27	108±57	20	105±53	27	117±61	20	112±43	27	108±46
Cyclophosphamide(oral or IV)	11	95±35	36	109±55	11	95±40	36	117±61	11	104±32	36	110±47
Cyclosporin A	3	75±10	44	108±52	3	96±19	44	113±59	3	98±19	44	110±45
Mycophenolate mofetil	8	96±38	39	107±53	8	121±49	39	110±59	8	113±51	39	109±43

┼ “*Flare*” is defined as increase in the SLEDAI score by 3 or more;

┼┼ “Ever” refers to use of immunosuppressants [prednisolone, hydroxychloroquine, azathioprine, cyclophosphamide (oral or IV), cyclosporin A and mycophenolate mofetil] since the diagnosis of SLE;

*p<0.05.

**Table 4:** Univariate analysis: the relationship between clinical characteristics, the use of drugs ever and the expression of NOD2 in SLE patients (categorical variables).

	NOD2 expression											
	Monocytes				Myeloid dendritic cells				Plasmacytoid dendritic cells			
	Yes		No		Yes		No		Yes		No	
	n		n		n		n		n		n	
**Clinical characteristics**												
Flare ┼	17	249±114	30	274±115	17	246±150	28	249±122	17	207±132	28	265±126
Elevated anti-dsDNA(>100 IU/ml)	21	234±117	26	290±107	20	226±147	25	265±118	20	210±140	25	271±120
Major organ involvement ≥1	32	241±104	15	316±121	30	240±146	14	264±98	30	232±137	14	289±117
**Clinical manifestations**												
Neuropsychiatric	4	228±155	43	168±111	4	221±184	41	250±128	4	239±140	41	243±131
Nephritis	27	237±106	20	302±117	25	232±141	20	269±104	25	222±135	20	279±119
Serositis	4	290±130	43	263±114	4	333±219	41	239±121	4	209±169	41	246±128
Hematologic	18	294±118	29	247±110	18	280±161	27	226±105	18	252±154	27	237±114
**Immunosuppressive** **therapy ever** ┼┼	**30**	**229**±**104** **	**17**	**329**±**105**	**28**	**211**±**143** ***	**17**	**308**±**83**	**28**	**192**±**130** ***	**17**	**325**±**79**
Prednisolone	**29**	**232**±**105** *	**18**	**318**±**114**	**27**	**212**±**146** **	**18**	**301**±**86**	**27**	**193**±**133** ***	**18**	**318**±**83**
** **Hydroxychloroquine	23	247±111	24	117±24	22	230±154	23	264±106	**22**	**201**±**143** **	**23**	**283**±**104**
Azathioprine	20	231±99	27	291±120	18	223±156	27	264±113	**18**	**199**±**133** *	**27**	**272**±**122**
Cyclophosphamide (oral or IV)	**11**	**198**±**77** *	**36**	**285**±**177**	9	201±102	36	259±137	9	186±100	36	257±134
Cyclosporin A	3	185±58	44	270±115	3	169±50	42	253±134	3	125±47	42	251±130
Mycophenolate mofetil	8	272±133	39	264±112	8	269±129	37	243±134	8	252±142	37	241±130

┼ “*Flare*” is defined as increase in the SLEDAI score by 3 or more;

┼┼ “Ever” refers to the use of immunosuppressants [prednisolone, hydroxychloroquine, azathioprine, cyclophosphamide (oral or IV), cyclosporin A and mycophenolate mofetil) since the diagnosis of SLE;

*p<0.05,

**p<0.01,

***p<0.001.

With regard to the role of immunosuppressants, the expression of NOD2 was significantly lower in CD4+ T, CD8+ T lymphocytes, monocytes, mDCs and pDCs of SLE patients ever treated with immunosuppressants when compared to those without immunosuppressants (Tables 3 and 4). Prednisolone use was associated with a significantly decreased expression of NOD2 in monocytes, mDCs and pDCs compared to the prednisolone naïve group (Table 4). Significantly decreased expression of NOD2 was found in patients treated with HCQ and AZA in pDCs compared with those who never been treated with HCQ and AZA (p<0.01) (Table 4). NOD2 expression in the monocytes was significantly lower in patients treated with CYC compared to the CYC naïve group (*p*<0.05). No differences were found in NOD2 expression between these patients who had or had not been treated with cyclosporin A or MMF (all *p*>0.05).

### Risk factors associated with NOD2 expression in SLE

In order to address whether the clinical characteristics; use of immunosuppressive therapy ever; current and cumulative dose of immunosuppressants and are independent predictors affecting the expression of NOD2, all of the potential explanatory variables with a *p*-value less than 0.05 identified in the univariate analysis [including disease duration, major organ involvement (≥1), current and cumulative dose of AZA, immunosuppressive therapy (prednisolone, HCQ, AZA and CYC) ever] were analyzed using linear regression analysis (Table 5). Ever use of immunosuppressive therapy was as an independent explanatory variable for downregulating the expression of NOD2 in CD8+ T [Adjusted coefficient (95%): −34.765 (−66.508 to −3.021); Adjusted R^2^: 0.191], monocytes [Adjusted coefficient (95%): −92.110 (−152.542 to −31.678); Adjusted R^2^: 0.163], mDCs [Adjusted coefficient (95%): −96.098 (−173.200 to −18.995); Adjusted R^2^: 0.108] and pDCs [Adjusted coefficient (95%): −132.403 (−203.118 to −61.689); Adjusted R^2^: 0.232] (all *p*<0.05). On the other hand, a longer disease duration was an independent risk factor for upregulating the expression of NOD2 in CD8+ T [Adjusted coefficient (95%): 3.152 (0.703 to 5.601); Adjusted R^2^: 0.121], CD19+ B [Adjusted coefficient (95%): 2.768 (0.836 to 4.700); Adjusted R^2^: 0.137] and monocytes [Adjusted coefficient (95%): 6.028 (1.366 to 10.691); Adjusted R^2^: 0.258] (all *p*<0.05) (Table 5).

**Table 5:** Independent risk factors associated with the expression of NOD2 in T and B lymphocytes, monocytes and DCs of SLE patients.

		Linear regression (stepwise selection) analysis				
		n	Risk factors	Adjusted coefficient (95%)	*P*-value	Adjusted R^2^
	**CD8+ T lymphocytes**	47	SLE duration, year	3.152 (0.703 to 5.601)	**0.013**	0.121
		47	Immunosuppressive therapy ever┼┼	−34.765 (−66.508 to −3.021)	**0.033**	0.191
	**CD19+ B lymphocytes**	47	SLE duration, year	2.768 (0.836 to 4.700)	**0.006**	0.137
**NOD2 expression**						
	**Monocytes**	47	Immunosuppressive therapy ever	−92.110 (−152.542 to −31.678)	**0.004**	0.163
		47	SLE duration, year	6.028 (1.366 to 10.691)	**0.012**	0.258
	**Myeloid dendritic cells**	45	Immunosuppressive therapy ever	−96.098 (−173.200 to −18.995)	**0.016**	0.108
	**Plasmacytoid dendritic cells**	45	Immunosuppressive therapy ever	−132.403 (−203.118 to −61.689)	**0.000**	0.232

┼┼ “Ever” refers to the use of immunosuppressants [prednisolone, hydroxychloroquine, azathioprine, cyclophosphamide (oral or IV), cyclosporin A and mycophenolate mofetil] since the diagnosis of SLE.

### Effects of NOD2 agonist (MDP) on the induction of cytokines from PBMC

In order to examine whether the NOD2 expression was functional, we exposed PBMC to specific NOD2 ligand MDP and investigated the *ex vivo* induction of cytokines using flow cytometry. As shown in Table 6, the basal productions of cytokines (IL-6, IL-8 and IL-10) were significantly increased in Group 1 and Group 3 compared with HCs (all *p*<0.05). Basal concentration of IL-10 was significantly higher in Group 2 compared to HCs, albeit significantly lower than Group 1.

**Table 6:** *Ex vivo* basal production and relative induction of cytokines from NOD2 ligand activated PBMC in various groups.

Cytokines			n	Basal production (IQR)(pg/10^5^ PBMCs)(Medium)	Relative induction (%) (IQR)(pg/10^5^ PBMCs)(MDP)
		Group 1	16	3.7 (2.9 to 4.4)	**212.0 (135.2 to 296.7)** Δ
**IL-1β**	SLE	Group 2	9	3.5 (3.3 to 3.7)	173.4 (156.8 to 244.1)
		Group 3	11	3.3 (3.1 to 3.7)	**217.2 (137.8 to 240.9)** §
	Healthy control		23	3.5 (3.1 to 4.1)	**153.9 (106.6 to 192.6)**
		Group 1	16	3.0 (2.6 to 3.7)	24.2 (21.0 to 33.8)
**TNF-α**	SLE	Group 2	9	2.6 (2.2 to 3.1)	19.6 (12.2 to 30.6)
		Group 3	11	2.9 (2.3 to 3.7)	23.5 (19.1 to 32.1)
	Healthy control		23	2.6 (2.0 to 3.2)	19.4 (16.9 to 25.2)
		Group 1	16	**12.2 (7.5 to 15.8)** Δ	18.9 (12.8 to 33.5)
**IL-6**	SLE	Group 2	9	10.8 (8.0 to 13.7)	12.1 (5.6 to 20.3)
		Group 3	11	**11.3 (9.1 to 16.4)** §§	23.3 (19.9 to 44.3)
	Healthy control		23	**8.1 (5.3 to 10.8)**	18.3 (14.7 to 19.6)
		Group 1	16	**929.8 (463 to 1263)** Δ	258.1 (216.9 to 298.5)
**IL-8**	SLE	Group 2	9	608.2 (407.9 to 809.7)	217.2 (137.8 to 240.9)
		Group 3	11	**727.2 (388.9 to 729.8)** §	212.0 (135.2 to 296.7)
	Healthy control		23	**552.8 (388.9 to 729.8)**	192.4 (117.1 to 219.3)
		Group 1	16	**6.8 (4.3 to 14.0)** &, ΔΔΔ	**57.4 (38.9 to 81.1) ^&&,^** Δ
**IL-10**	SLE	Group 2	9	**4.8 (3.8 to 5.3)** ΦΦ	**20.9 (14.4 to 38.5)** Φ
		Group 3	11	**5.2 (5.0 to 5.6)** §§	40.5 (27.0 to 59.1)
	Healthy control		23	**2.4 (2.3 to 2.8)**	**33.6 (19.4 to 46.8)**

Culture supernatant was obtained from PBMCs cultured with medium or NOD2 ligand (Muramyl dipeptide, MDP) for 24 hours. The basal production (pg/ml) and relative induction (%) of cytokines (IL-1β, TNF-α, IL-6, IL-8 and IL-10) by PBMCs were analyzed by flow cytometry. Numerical data are expressed as median (interquartile range, IQR). Group 1) Patients with inactive disease (SLEDAI <4) who were never treated with immunosuppressants since the diagnosis or within the past 10 years, whichever longer; Group 2) Patients with inactive disease (SLEDAI <4) who had received or are currently on immunosuppressants; Group 3) Patients with active disease (SLEDAI >4) who had received or are currently on immunosuppressants.

& *p*<0.05, comparing between Group 1 and Group 2;

Δ *p*<0.05,

ΔΔΔ *p*<0.001, comparing between Group 1 with healthy control subjects;

Φ *p*<0.05,

ΦΦ *p*<0.01, comparing between Group 2 with healthy control subjects;

§ *p*<0.05,

§§ *p*<0.01 comparing between Group 3 with healthy control subjects.

As shown in Table 6, the relative induction (%) of cytokines (IL-1β and IL-10) upon NOD2 ligand (MDP) stimulation from PBMC was significantly increased Group 1 compared with HCs (*p*<0.05). The relative induction (%) of IL-1β in response to MDP was increased in Group 3 compared to HCs (*p*<0.05). An observable diminished relative induction (%) of IL-10 was observed in Group 2 compared to Group 1 and HCs (*p*<0.05). No significant differences were observed for the relative induction (%) of TNF-α, IL-6 and IL-8 from PBMCs upon the activation by NOD2 ligand between the four groups (all *p*>0.05).

## Discussion

Bacterial (*Staphylococcus aureus*, *Streptococcal pneumonia*, *Escherichia coli a*nd *Pseudomonas aeruginosa*) [30] and viral infections (cytomegalovirus, Epstein-Barr virus, parvovirus B19 and papillomavirus) have been suggested to be pathogenic triggers for the development or exacerbation of SLE either through persistent infection or the presence of remnants of pathogens [31], [32]. Recognition of these microorganisms is mediated, at least in part, by TLRs, and TLR-induced pathways are responsible for the induction of proinflammatory mediators [33], [34]. In addition to TLRs, *Pseudomonas aeruginosa* and *Streptococcal pneumonia* can also be recognized by NOD1 and NOD2, respectively [35]. Activation of NOD1 and NOD2 has been shown to stimulate the cytokine production and neutrophil recruitment [36], and mediate TLR-induced cytokine production [5], [15], respectively. Apart from the putative link between the genetic variants of NOD2 and SLE [18], [19], [20], [21], [22], [23], little is known about the expression and function of NOD2 in SLE.

NOD2 is expressed in intestinal epithelial paneth cells, lymphocytes and myeloid cells such as monocytes, macrophages and DCs that are in direct contact with microbial organisms, whereas NOD1 is expressed in non-myeloid cells such as epithelial cells and fibroblasts [37], [38]. When the NOD2 gene functions normally, it codes for a cytosolic innate receptor which is able to sense PGNs from Gram-positive and negative bacteria and to trigger signaling pathway mediated pro-inflammatory and anti-bacterial response [39]. Van *et al.* demonstrated that the T helper (Th)17-mediated, antibacterial immunity in humans is orchestrated by DCs upon sensing of bacterial NOD2-ligand MDP [40]. In patients with chronic inflammatory diseases, aberrant expression and function of NOD2 has been reported mainly in inflammatory bowel disease and other inflammatory disorders [6], [16], [17]. Recent studies have demonstrated that the mRNA[16] and protein[6] of NOD2 were expressed in synovial tissue samples from RA patients, and NOD2 activation in synovial fibroblasts leads to the expression of proinflammatory cytokines and matrix-degrading enzymes via p38 mitogen-activated protein kinases (MAPK) and NF-κB signaling pathways [6]. In agreement with previous studies, we observed a significantly higher expression of NOD2 in monocytes and pDCs in immunosuppressive therapy naïve SLE patients (Group 1) compared with HCs. Inflammatory burden as reflected by the disease duration played a role as an independent explanatory variable for upregulating the expression of NOD2 in monocytes.

Cytokines play an important and diverse role in the pathogenesis of SLE, and their balance determines disease activity [41], [42]. Proinflammatory cytokines such as IL-1β, IL-6, IL-8 and TNF-α are found to possess potent stimulatory effects on T, B lymphocytes, natural killer (NK) cells, and neutrophils in increasing the biosynthesis of prostaglandins and acute-phase proteins [43], whereas Th2 cells produce anti-inflammatory cytokine, IL-10, to modulate immunity to extracellular parasites [44]. Elevated basal production of cytokines including TNF-α, IL-6, IL-8 and IL-10, both in serum and culture, has been reported in SLE patients compared with healthy controls [45], [46], [47]. Our results concurred with previous studies showing that the basal production of cytokines (IL-6, IL-8 and IL-10) was significantly increased in immunosuppressive naïve patients (Group 1) and patients with active disease despite immunosuppressive therapy (Group 3) compared with HCs, implying that the imbalance between proinflammatory (IL-6 and IL-8) and anti-inflammatory (IL-10) cytokines may trigger the inflammatory process in SLE.

In order to confirm whether the NOD2 protein was functional, NOD2 agonist (MDP)-stimulated production of pro- and anti-inflammatory cytokines from SLE patients and HCs were measured. Production of IL-1β has been reported upon stimulation with MDP [48], [49], while IL-1β blockade profoundly inhibits MDP-induced cytokine production in macrophage, demonstrating a key role of IL-1β in NOD2-mediated inflammation[49]. Consistent to the previous finding, upon stimulation with NOD2 agonist, our data showed an observable increased relative induction (%) of IL-1β upon MDP stimulation in SLE patients (Groups 1 and 3) compared to controls. IL-1 is one of the most pleiotropic pro-inflammatory and immunostimulatory cytokines. Overproduction of IL-1 has been shown to be involved in the pathogenicity of various autoimmune inflammatory diseases, including SLE. In an experimental model, mice deficient in IL-1β developed lower levels of anti-dsDNA antibodies, milder disease manifestations and reduced cytokine cascade that is the characteristics of overt experimental SLE [50]. Spontaneous release of IL-1 from SLE monocytes has been reported as increased in many, but not all studies [41], [51], [52]. Therefore, the proinflammatory state resulting from the increased expression of NOD2 in the immunosuppressive naive SLE patients, together with previous reports regarding the increase in the expression of TLRs [7], underscores the importance of the activation of innate immune mechanisms in SLE. Data from our group as well as others provided evidence which may suggest that certain bacterial/viral components from an initial infection may lead to the activation of innate immune receptors, which provokes an inflammatory reaction and contribute towards the initiation of the pathogenic process in SLE. Further functional, signaling and genetic work will need to be performed to investigate whether higher level of cytokines are a result of NOD2-mediated flare in SLE, or whether the pro- or anti-inflammatory cytokines play a role in initiating and perpatuating the NOD2-mediated inflammatory process.

We have demonstrated that immunosuppressive therapy may play a role as an independent explanatory variable associated with the decreased expressions of NOD2 in CD8+ T, monocytes, mDCs and pDCs. Emerging evidence has reported that derivatives and small molecule analogues of chloroquine and quinacrine suppress the over-activation of immune response.[53], [54] These anti-malarial agents[55] (chloroquine, HCQ and quinacrine) used for the treatment of immune-mediated inflammatory disorders (IMID) such as SLE, are antagonists of TLR-9 and to a lesser extent, TLR-7 and -8 [54]. Similarly, high dose glucocorticosteroids could also reduce the *in vivo* TLR responses by 70–90% [56]. The effects of TLR antagonists on NOD2 expression in SLE patients have not been well elucidated, nonetheless, a synergistic effect of NODs and TLRs has been reported in other diseases [6], [57], [58]. Whether immunosuppressive therapy may have a direct effect on downregulating the expression of NOD2 in SLE patients, or indirectly through the interaction between NOD2 and TLRs in SLE patients need further investigation.

In the present study, the basal concentration of IL-10 was significantly lower in patients with inactive disease receiving immunosuppressive therapy compared to those without immunosuppressants. Furthermore, the use of immunosuppressant was associated with an observable diminished relative induction (%) of IL-10 upon stimulaiton with MDP in SLE patients with inactive disease. IL-10 is an important immunoregulatory cytokine that inhibits T cell function by suppressing the expression of proinflammatory cytokines such as TNF-*α*, IL-1, IL-6, IL-8, and IL-12 [59]–[60]. It also inhibits antigen presenting cells by downregulating major histocompatibility complex class II (MHC-II) and B7 expression [61]. In addition to these inhibitory actions, IL-10 promotes B-cell-mediated functions, enhancing survival, proliferation, differentiation and antibody production [62]. Hence, increased production of IL-10 could thus explain B cell hyperactivity and autoantibody production, two main features of the immune dysregulation in SLE. In fact, elevated levels of this molecule have been currently reported in SLE patients, frequently associated with the indicators of disease activity [63], [64]. NOD2 mutation in CD patients results in the suppression of the production of IL-10 [65]. Here, we hypothesize that part of the beneficial effect of immunosuppressive therapy may be mediated by the downregulation of NOD2, resulting in a redution of the basal and MDP induced production of IL-10 in SLE patients. Further studies exploring the mechanisms of how immunosuppressant regulate NOD2 expresssion and function in SLE will be warranted.

Failure to induce an effective cellular immune response because of inefficient activation of innate immunity and ineffective priming of the adaptive immune response in patients with lupus may lead to an increased prevalence as well as persistence of any infection. In patients with CD, dysfunctional NOD2 variants in the intestinal epithelial and phagocytic cells results in the deficiencies in epithelial-barrier function which subsequently lead to increased bacterial invasion and inflammation at intestinal sites. Other study has demonstrated that NOD2-deficient mice infected orally with *L. monocytogenes* had a significantly greater bacterial burden in liver and spleen than wild-type mice [66]. NOD2-deficient mice have impaired resistance to mycobacterium tuberculosis infection through defective innate and adaptive immunity [67]. Our group also reported a higher prevalence of mycobacterial tuberculosis infection in patients with SLE [68], and there are several reports suggesting that mycobacterium tuberculosis infection precipitates SLE in patients from endemic areas [69]. Whether decreased NOD2 expression may contribute towards the induction and reactivation of chronic inflammation by mycobacterial infection in patients with SLE deserves further study. On the other hand, apart from the direct immuno-modulating effects, the use of immunosuppressive agents may increase the risk of infections in SLE patients. Viral infections have been reported to down-regulate innate immune receptors in the host resulting in persistent infection, e.g. human papillomavirus virus (HPV) type 16 infection has been reported to down-regulate TLR 9 pathway promoting persistent infection in the host [70]. Similarly, chronic hepatitis C infection leads to a decrease in expression and functional impairment of TLR2 [71]. Whether bacteria could also make use of this immune evasion strategy in order to escape NOD2 recognition in patients with SLE would deserve further studies.

The main limitation of this study is that we only studied the function of NOD2 in the PBMCs. Further studies of the differential function of NOD in different immune cell types, detailed cellular regulatory mechanisms of NOD2 activation and the interaction among NOD2, TLRs and the effects of the pro- and anti-inflammatory cytokines in SLE would be worth studying. Although our findings have speculated that the immunosuppressive therapy may be as important factor in down-regulating the expression of NOD2, we did not explore the drug effect on different cell types both *in vivo* and *in vitro*. It would be of interest to further explore the mechanism in regulating NOD2 expression and function by immunosuppressive agents including prednisolone, TLR antagonists and other immunosuppressive therapies in SLE. Furthermore, a future prospective study with a larger sample size followed for a longer period of time is warranted to study the risk factors affecting the expression of NOD2 in SLE patients.

In conclusion, exposure to bacterial conserved molecular pattern may increase the expression of NOD2 in the monocytes of immunosuppressant naïve patients, which can subsequently lead to aberrant activation of PBMCs, resulting in the production of proinflammatory cytokines, implicating the innate immune response for extracellular pathogens in the immunopathological mechanisms in SLE. On the other hand, immunosuppressive therapies may downregulate the NOD2 expression in CD8+ T lymphocytes, monocytes, mDCs and pDCs in SLE patients which subsequently reduce IL-10 production, contributing towards the regulation of immunopathological mechanisms of SLE, at the expense of increasing the risk of concurrent bacterial infection.
